# Predictive significance of *FGFR4* p.G388R polymorphism in metastatic colorectal cancer patients receiving trifluridine/tipiracil (TAS-102) treatment

**DOI:** 10.1186/s12967-024-05184-w

**Published:** 2024-04-22

**Authors:** Alessandro Ottaiano, Mariachiara Santorsola, Monica Ianniello, Anna Ceccarelli, Marika Casillo, Francesco Sabbatino, Nadia Petrillo, Marco Cascella, Francesco Caraglia, Carmine Picone, Francesco Perri, Roberto Sirica, Silvia Zappavigna, Guglielmo Nasti, Giovanni Savarese, Michele Caraglia

**Affiliations:** 1 Istituto Nazionale Tumori Di Napoli, IRCCS “G. Pascale”, Via M. Semmola, 80131 Naples, Italy; 2 Centro Polidiagnostico Strumentale Srl, AMES, 80013 Naples, Italy; 3 Medical Oncology, Fondazione Policlinico Universitario Agostino Gemelli IRCCS- Università Cattolica del Sacro Cuore, Rome, Italy; 4 Department of Medicine, Surgery and Dentistry, University of Salerno, 84081 Baronissi, Italy; 5 Department of Precision Medicine, University of Campania “L. Vanvitelli”, Via L. de Crecchio, 7, 80138 Naples, Italy; 6 Laboratory of Precision and Molecular Oncology, Institute of Genetic Research, Biogem Scarl, Ariano Irpino, Italy

**Keywords:** TAS-102, FGFR4, Colorectal cancer, NGS

## Abstract

**Background:**

TAS-102 (Lonsurf^®^) is an oral fluoropyrimidine consisting of a combination of trifluridine (a thymidine analog) and tipiracil (a thymidine phosphorylation inhibitor). The drug is effective in metastatic colorectal cancer (mCRC) patients refractory to fluorouracil, irinotecan and oxaliplatin. This study is a real-world analysis, investigating the interplay of genotype/phenotype in relation to TAS-102 sensitivity.

**Methods:**

Forty-seven consecutive mCRC patients were treated with TAS-102 at the National Cancer Institute of Naples from March 2019 to March 2021, at a dosage of 35 mg/m^2^, twice a day, in cycles of 28 days (from day 1 to 5 and from day 8 to 12). Clinical-pathological parameters were described. Activity was evaluated with RECIST criteria (v1.1) and toxicity with NCI-CTC (v5.0). Survival was depicted through the Kaplan-Meyer curves. Genetic features of patients were evaluated with Next Generation Sequencing (NGS) through the Illumina NovaSeq 6000 platform and TruSigt™Oncology 500 kit.

**Results:**

Median age of patients was 65 years (range: 46–77). Forty-one patients had 2 or more metastatic sites and 38 patients underwent to more than 2 previous lines of therapies. ECOG (Eastern Cooperative Oncology Group) Performance Status (PS) was 2 in 19 patients. The median number of TAS-102 cycles was 4 (range: 2–12). The most frequent toxic event was neutropenia (G3/G4 in 16 patients). There were no severe (> 3) non-haematological toxicities or treatment-related deaths. Twenty-six patients experienced progressive disease (PD), 21 stable disease (SD). Three patients with long-lasting disease control (DC: complete, partial responses or stable disease) shared an *FGFR4* (p.Gly388Arg) mutation. Patients experiencing DC had more frequently a low tumour growth rate (*P* = 0.0306) and an *FGFR4* p.G388R variant (*P* < 0.0001). The *FGFR4* Arg388 genotype was associated with better survival (median: 6.4 months) compared to the Gly388 genotype (median: 4 months); the HR was 0.25 (95% CI 0.12- 0.51; P = 0.0001 at Log-Rank test).

**Conclusions:**

This phenotype/genotype investigation suggests that the *FGFR4* p.G388R variant may serve as a new marker for identifying patients who are responsive to TAS-102. A mechanistic hypothesis is proposed to interpret these findings.

**Graphical Abstract:**

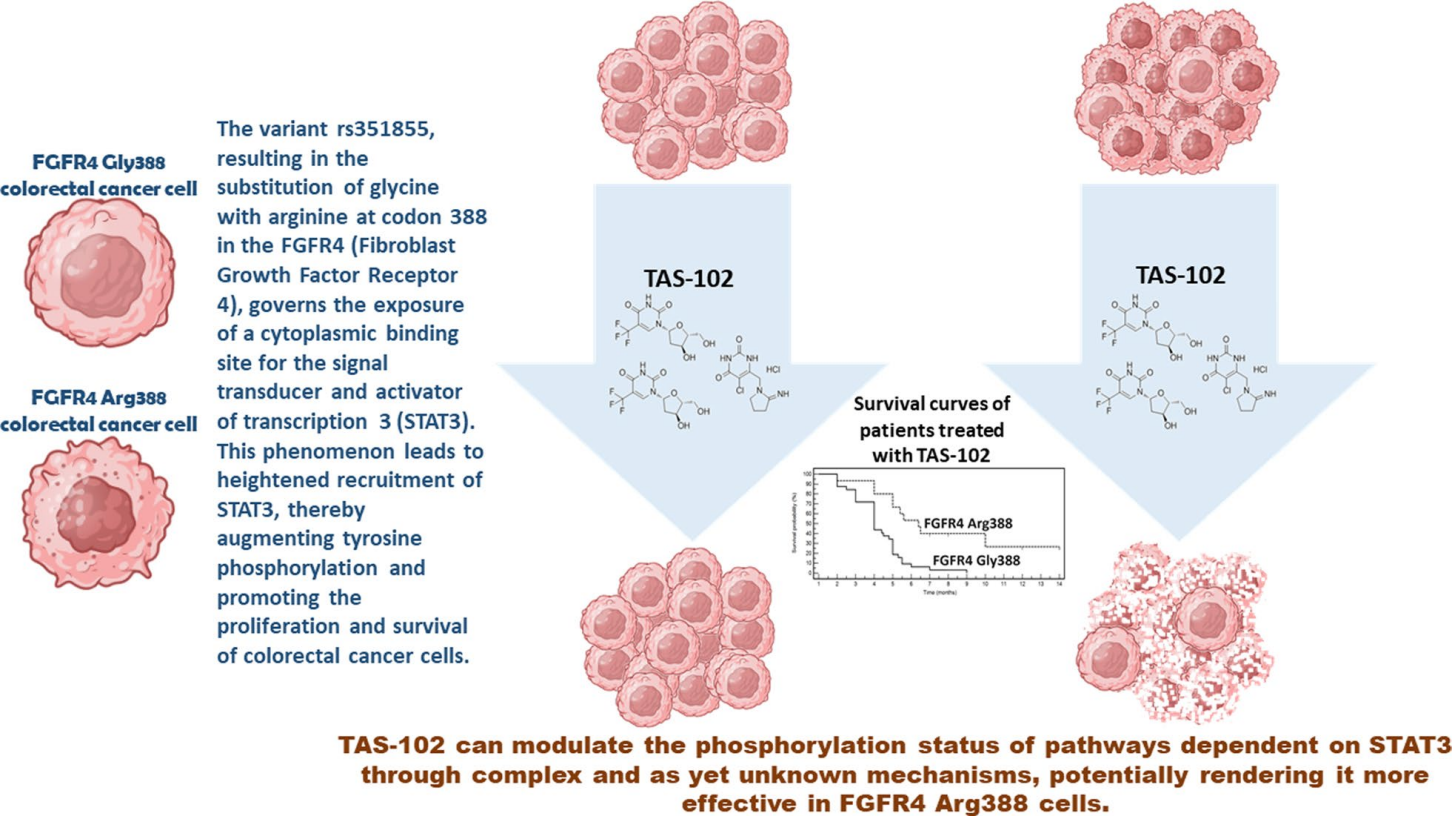

## Background

Fluoropyrimidines (FPDs) represent a milestone in the treatment of colorectal cancer (CRC) both in the adjuvant and metastatic settings. They inhibit the thymidylate synthetase (TS), a fundamental enzyme in the synthesis pathway of pyrimidine nucleotides [1]. 5-flurouracil (5-FU) and capecitabine (pro-drug of 5-FU) belong to this category and constitute an essential component of chemotherapy regimens, in combination with irinotecan and/or oxaliplatin, currently used in metastatic CRC (mCRC) in association with new biologic drugs (bevacizumab, aflibercept, panitumumab, cetuximab) [2]. TAS-102 (Lonsurf^®^) is a combination of two drugs: trifluridine (a thymidine analogue) and tipiracil (a thymidine phosphorylation inhibitor) [3]. The triphosphate form of trifluridine (TAS-102) is incorporated into the DNA determining its anti-tumor effects while tipiracil prevents the degradation of TAS-102 by maintaining adequate plasma levels. It is administered at a dosage of 35 mg/m^2^, twice a day, in cycles of 28 days, from day 1 to 5 and from day 8 to 12 [4, 5]. In previous studies, the drug has been shown to be effective in mCRC patients refractory to fluorouracil, irinotecan and oxaliplatin [6, 7] determining an increase median patients’ survival of approximately 2 months (with a significant reduction of death risk by 32%) *versus* placebo and an acceptable toxicity profile. The benefit was evident and significant in all patients’ subgroups (ECOG PS 0 *vs* 1, *KRAS* mutated *vs* not mutated, right vs left colon, young *vs* elderly, Asian *vs* Caucasian, poly-metastatic *vs* oligo-metastatic disease, etc*.*). The most frequent toxic events were mainly haematological (neutropenia) while non-haematological toxicities were not significantly higher than placebo. Due to its good risk/benefit ratio, TAS-102 is increasingly used in the treatment of mCRC after failure of previous therapies (including 5-FU, oxaliplatin and irinotecan).

Recently, genetic assessments have provided a useful tool to optimize treatment of mCRC patients including analysis of genetic variants of: 1. *DPD* (dihydropyrimidine dehydrogenase) and *UGT1* (uridine diphosphate glucuronosyl-transferase), causing reduced/abrogated metabolism and risk of extremely severe toxicities from FDPs [8] and irinotecan [9], respectively, 2. *RAS* (RAt Sarcoma viral oncogene homolog) and *BRAF* (v-raf murine sarcoma viral oncogene homolog B), determining a constitutive activation of EGFR-dependent proliferative pathway of MAP kinases and consequent unresponsiveness to anti-EGFR blocking agents (panitumumab and cetuximab) [10], 3. micro-satellites status (MSS), short and repetitive predominantly non-coding DNA sequences which can accumulate alterations (becoming unstable) [11], 4. mismatch repair (MMR) genes (including *MSH2*, *MLH1*, *MSH6*, *PMS2*) [12]. In the last two cases, the tumours are more prone to mutate and accumulate neo-antigens and, consequently, to respond to immunotherapy (immune check-point inhibitors) [13].

In this study, we present a real-world, consecutive, two-year analysis of TAS-102 administration, investigating the genomic profiles of patients who benefited clinically. Our aim is to generate hypotheses regarding potential biomarkers and molecular factors associated with TAS-102 responsiveness.

## Methods

### Patients, treatments and follow-up

Patients were enrolled at the SSD-Innovative Abdominal Metastasis Therapies of the Department of Abdominal Oncology of the National Cancer Institute of Naples from March 2019 to March 2021. We reported the following clinical-pathological parameters: age, gender, histological characteristics, tumor site, PS (Performance Status) according to ECOG (Eastern Cooperative Oncology Group), disease sites, previous treatments.

The first- and second-line treatments were carried out in accordance with the ESMO guidelines [14]. No BRAF mutated patients of this cohort received “on-label” encorafenib for reasons of timing approval of the Italian regulatory authorities and clinical appropriateness. All patients had cytologically and/or histologically confirmed CRC. TNM staging (according to AJCC) was performed with total-body Computerized Tomography (CT) before the start of treatments (regardless of the line). Considering the real-word nature of this cohort, CT scan monitoring was not centralized, and it was performed every three months (± 14 days) or anticipated in case of clinically suspected progression of disease. Other examinations (ultrasound, MRI, PET, RX, etc.) were carried out at the discretion of the reference oncologist and in relation to specific clinical questions. TAS-102 was administered *per os* at a dose of 35 mg/m^2^ twice daily on days 1–5 and days 8–12 in a 28-day cycle. Response to treatments was assessed according to RECIST v1.1 criteria [15]. Disease Control (DC) rate was defined as the percentage of patients who have achieved complete response (CR), partial response (PR) and stable disease (SD). Duration of DC was the time elapsing from the date of TAS-102 start to the date of the first progressive disease (PD) documentation. Treatment toxicity has been reported in this article according to CTC v5.0 criteria (www.eortc.be/services/doc/ctc). According to the internal policies of our Institute, the institutional review board approval was not required for the retrospective analysis of this clinical cohort. All patients signed a written informed consent before treatment administration and molecular assessments.

### NGS assessment and genetic data reporting

Gene mutations were assessed on formalin-fixed paraffin-embedded (FFPE) of metastatic tumor tissues if available (biopsies or tumour resections). DNA was extracted from three 10 µm FFPE sections by means of the MGF03-Genomic DNA FFPE One-Step Kit, according to the manufacturer’s protocol (MagCore Diatech). DNA quality was evaluated in triplicate with the FFPE QC Kit according to the manufacturer’s protocol (Illumina, San Diego, USA). Libraries were prepared thorough the TruSigt TMOncology 500 kit. Sequencing was performed on an Illumina NovaSeq 6000 (San Diego, USA) platform. The assay detects small nucleotide variants (SNVs), indels, splice variants and immunotherapy biomarkers in 523 cancer-relevant genes. An Illumina TruSigth Oncology 500 bioinformatics pipeline was applied to analyse the sequencing results, as previously reported [16, 17]. The prioritization of variants was done according to a four-tiered structure, adopting the joint consensus recommendation by AMP/ACMG [18]. Missense genetic variants were reported through the p. notation (p. followed by the new amino acid which replaces the wild-type amino acid) and Venn-Diagrams were applied in order to depict intersections among them (shared and private mutations) in different patients.

Tumor Mutational Burden (TMB) was estimated by counting all coding alterations within the targeted genomic region [19]. A robust and precise model was utilized for predicting the MSI phenotype, based on TMB dichotomization into low/intermediate (0–19 mutations/mb) and high (≥ 20 mutations/mb) categories, achieving both positive and negative predictive values of approximately 99% [19, 20].

### Tumour growth rate assessment

The tumour growth rate (TGR) was calculated with the algorithm 100x(STL_progression_-STL_preceding_)/STL_preceding_/T) were STL is the sum of target lesions (STL_progression_: at progression; STL_preceding_: immediately preceding STL_progression_) and T is the time elapsed between these evaluations. TGRs was classified in low TGR and high TGR as previously reported [21]. Briefly, low TGR was < 0.33%/day and no new metastatic sites, high TGR was ≥ 0.33%/day or emergence of new metastatic sites.

### Setting of LDH values

The cut-off value to discriminate between high *vs* low LDH (Lactic DeHydrogenase) serum levels was chosen as previously reported [22]. It was settled at 1.2 × ULN (Upper Limit Normal) U/L: 270 U/L.

### Statistical analysis

Data were extracted from an internal electronic database prospectively updated. To avoid negative prognostic interferences, patients with PS ECOG > 2, age > 80 years, and an oncologist-estimated life expectancy < 3 months were excluded. Survival was intended as the time elapsed from the first administration of TAS-102 until death or the last available follow-up and was depicted with Kaplan-Meyer curves. The statistical significance of time-to-outcome divergences according to selected prognostic factors was studied with the Log-Rank test (P < 0.05 was considered statistically significant). The reported Hazard Ratios (HRs) are the estimate of the survival probability, and they can be interpreted as the instantaneous relative risk of death, at any time, for an individual. 95% confidence intervals (CI) of HR are also reported. Analyses of time-to-outcome are predominantly descriptive and no attempts to other statistical inferences have been made. Associations between *FGFR4* p.G388R, TGR, basal CEA, NLR, LDH and response to TAS-102 were evaluated by χ2 test. *P* < 0.05 was considered statistically significant. Statistical analysis was performed using the MedCalc^®^ 20.011 and Excel software.

## Results

### Clinico-pathological characteristics of clinical cohort

Forty-seven consecutive patients treated with trifluridine/tipiracil for metastatic CRC were enrolled. Clinico-pathological characteristics are reported in Table 1. The median age was 65 years (range: 46–77). Twenty-seven were male, 20 female. Four patients had a mucinous histology. The site of the primary tumor was right colon in 25, left in 22. Nineteen patients started TAS-102 therapy with PS ECOG 2. Six patients had single organ involvement (5, liver; 1, peritoneum), 41 had lesions in multiple sites (liver, lungs and lymph nodes, 13; liver and lungs, 9; liver and peritoneum, 8; liver, lymph nodes and bones, 4; lungs, liver, lymph nodes and peritoneum, 4; lungs and peritoneum, 2; peritoneum, 1). *KRAS* oncogene was mutated (mut*KRAS*) in 13 patients (27%) and wild-type (wt*KRAS*) in 34. *BRAF* p.V600E mutation was present in 4 patients (9%). Thirty-eight patients received more than two previous treatment lines. All patients were refractory to oxaliplatin- and irinotecan-based schedules. Twenty-eight patients were treated with regorafenib before TAS-102. Sixteen patients experienced objective responses (complete or partial according to RECIST v1.1) to previous first-line therapy (Table 2).

**Table 1:** Clinico-pathological characteristics of patients

Variable	No	%
Age		
Median (year)	65	
Range (year)	46–77	
Gender		
Male	27	57.4
Female	20	42.6
Histology		
Classical adenocarcinoma	43	91.5
Mucinous adenocarcinoma	4	8.5
Side		
Right	25	53.2
Left	22	46.8
PS ECOG		
0	4	8.5
1	24	51.1
2	19	40.4
No. of metastatic sites		
1	6	12.8
≥ 2	41	87.2
*K- or N-RAS*		
Mutated	13	27.7
Wild-type	34	72.3
*BRAF*		
Mutated	4	8.5
Wild-type	43	91.5
*FGFR4 p.G388R*		
No	32	68.1
Yes	15	31.9

**Table 2:** Previous treatments

Characteristic	No	%
No. of previous treatment lines		
2	9	19.2
3	19	40.4
4	19	40.4
Regorafenib before TAS-102		
Yes	28	59.6
No	19	40.4
Response to first-line chemotherapy		
CR	1	2.1
PR	15	31.9
SD	22	46.8
PD	9	19.2

### Toxicity and response

**Fig. 1:**
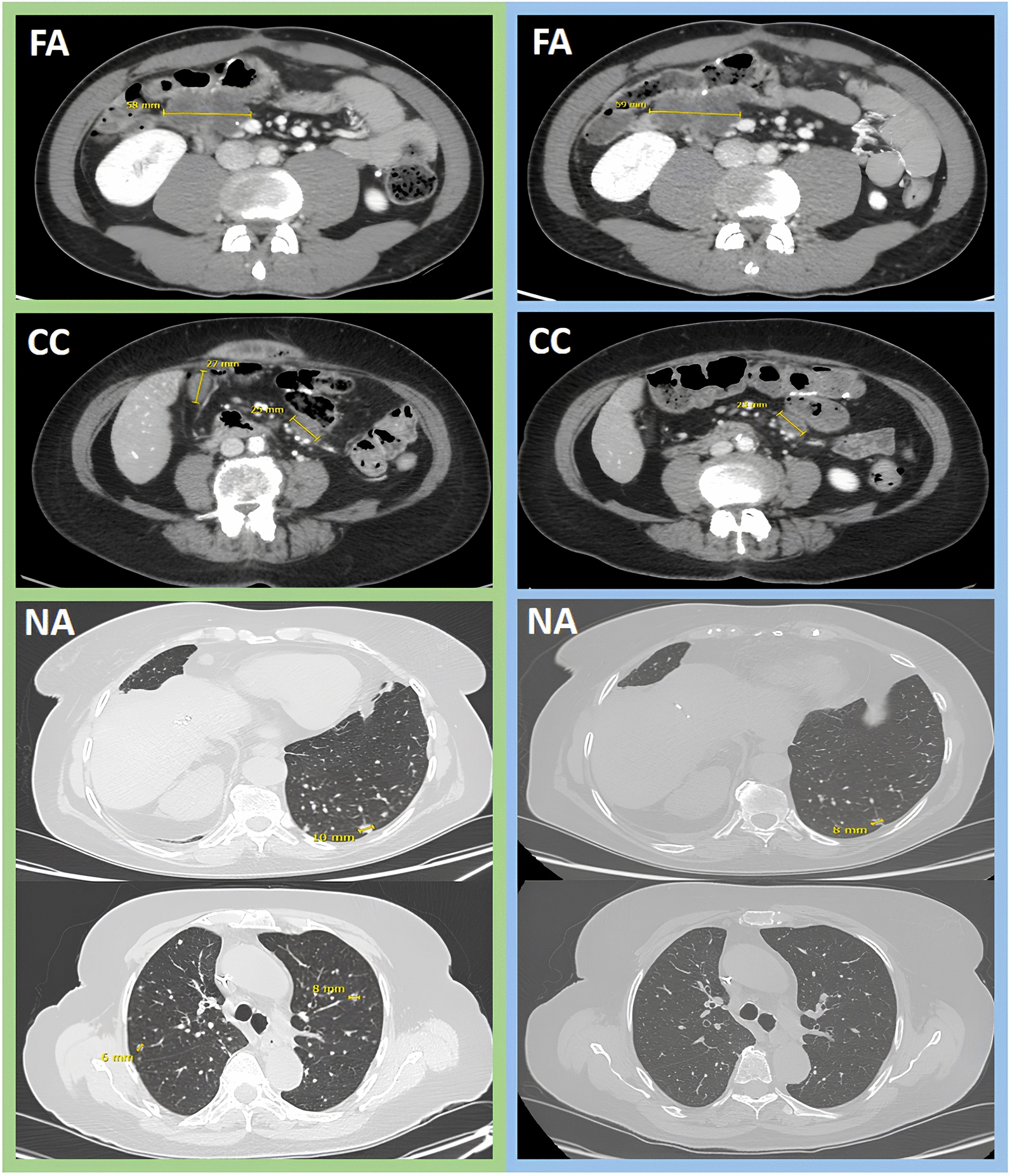
Representative computed tomography (CT) restaging images depict FA, CC, and NA patients with long-lasting disease control (FA, CC, and NA represent patients' initials). Baseline CT scans at TAS-102 treatment initiation are outlined in green frames, while CT scans at the last available restaging (third for FA and CC patients, second for NA patients) are framed in blue. Measurable lesions in the peritoneum (FA: 58 mm vs 59 mm), abdominal lymph nodes (CC: 22 mm vs 0 mm; 25 mm vs 23 mm), and lungs (NA: 10 mm vs 8 mm, 6 mm and 8 mm vs 0 mm and 0 mm) are delineated by their longest diameters, with measurements in millimeters reported within the images

The median number of administered cycles (1 cycle = 28 days) was 4 (range: 2–12). The most frequent toxic event attributable to the administration of TAS-102 was neutropenia (G3/G4 in 16 patients). There were no severe (> 3) non-haematological toxicities or treatment-related deaths (Table 3). Nine patients started therapy at reduced doses (− 5 mg/m^2^/dose). A dose adjustment (reduction of at least -5 mg/m^2^/dose) was applied in 29 patients (Table 4) during treatment course. A radiologic re-evaluation of the disease was carried out in 33 patients. Fifteen patients did not undergo to instrumental restaging because of a clear clinical evidence of progressive disease and rapid deterioration of general conditions. The outcome of these patients is formally indicated as PD. No responses were recorded according to RECIST v1.1 criteria. Eleven patients showed instrumental PD, 21 had SD (DC rate: 44.7%). Figure 1 shows representative CT restagings in patients experiencing long-lasting DC.

**Table 3:** Hematologic and non-hematologic toxic events

Toxicity	No	%
Alopecia		
G1/G2	8	17.0
G3	2	4.3
Anemia		
G1/G2	21	44.7
G3	5	10.6
AST/ALT increase		
G1/G2	8	17.0
G3	2	4.3
Fatigue		
G1/G2	26	55.3
G3	8	17.0
Diarrhea		
G1/G2	22	46.8
G3	4	8.5
Nausea/Vomiting		
G1/G2	18	38.3
G3	1	2.1
Neutropenia		
G1/G2	18	38.3
G3	14	29.8
G4	2	4.2
Stomatitis		
G1/G2	18	38.3
G3	2	4.2
Thrombocytopenia		
G1/G2	9	19.1
G3	2	4.2

**Table 4:** Compliance to TAS-102 treatment

Dose reductions	No	%
Ab initio (− 5 mg/m^2^/dose)	9	19.1
During treatment	29	61.7
− 5 mg/m^2^/dose	12	
− 10 mg/m^2^/dose	8	
− 15 mg/m^2^/dose	9	
No dose reduction	9	19.1

### Genetic sharing in tumours of patients experiencing long-lasting DC to TAS-102 therapy

**Fig. 2:**
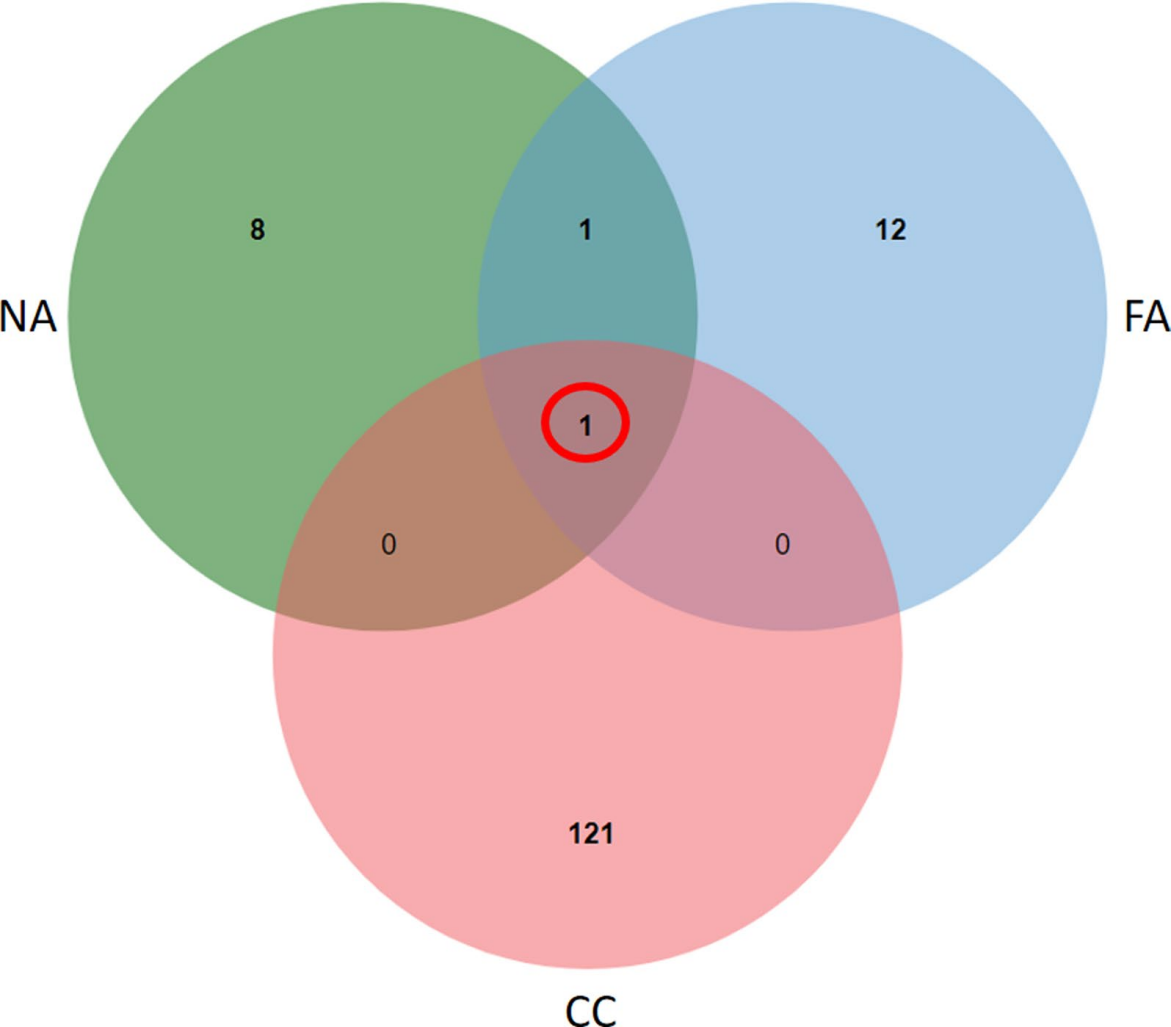
Venn diagram illustrating the coding genetic variant shared among patients exhibiting long-lasting disease control with TAS-102 (FA, CC, and NA represent patients' initials). The sole shared genetic variation (highlighted in red) is *FGFR4* p.G388R. DNA extraction was performed from formalin-fixed and paraffin-embedded (FFPE) primary colorectal cancer tissue specimens. Libraries were prepared using the TruSigtTMOncology 500 kit, which assesses 523 cancer-relevant genes, and sequenced on an Illumina NovaSeq 6000 platform. Sequences were aligned to the human reference genome GRCh37 using the Burrows–Wheeler Aligner tool (for further details, refer to the Methods section)

Since the genetic characteristics of tumors can be linked to specific clinical behaviors, including responses to treatments and the extent of time to outcomes, we selected three patients who experienced the longest disease control durations with TAS-102 (10, 12, and 14 months of disease control). We then correlated their genetic landscape, analyzed through NGS. The results are depicted in Venn Diagrams in Fig. 2. Interestingly, these patients shared only one genetic variant, *FGFR4* p.G388R.

### Time-to-outcome

**Fig. 3:**
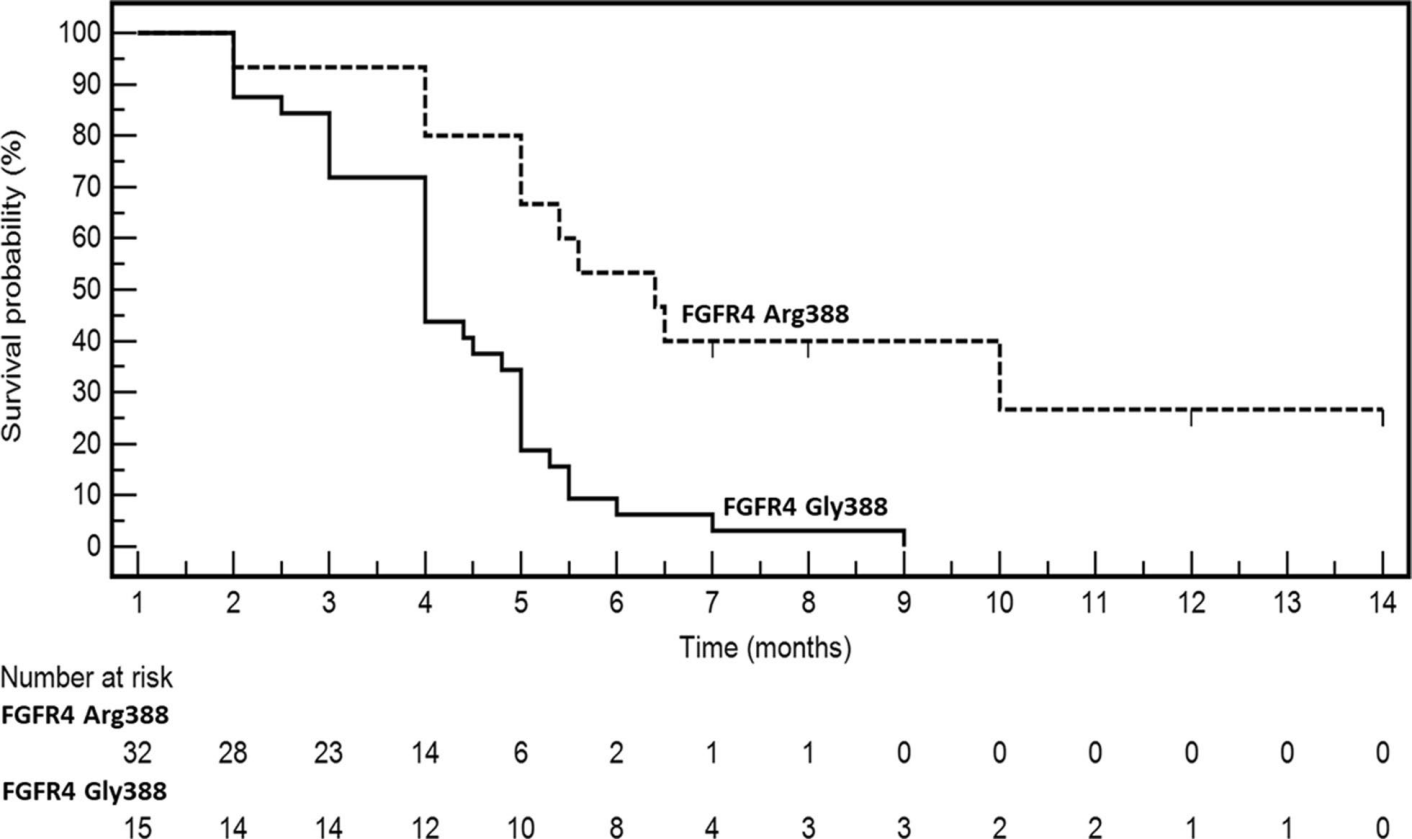
Kaplan–Meier survival curves depict patient outcomes from the initiation of TAS-102 therapy until death, stratified by the presence or absence of the rs351855 *FGFR4* polymorphism. Time is represented on the x-axis, while survival probability is depicted on the y-axis. The number of patients at risk is reported below the figure, with data categorized based on *FGFR4* polymorphism status

OS was considered the best time-to-outcome descriptor since CT scans were not centralized and 15 patients did not undergo instrumental restaging. The median survival of the entire cohort from TAS-102 start was 4.8 months; at last follow-up, there were 42 events (deaths). Since *FGFR4* p.G388R was the only genetic variant shared by long-lasting DC patients, its prognostic impact on survival of the entire clinical cohort of metastatic CRC patients treated with TAS-102 was studied (Fig. 3). All primary tumours were genetically characterized. Fifteen patients had the *FGFR4* p.G388R variant (31.9%). Median survival of patients with the *FGFR4* Arg388 genotype (events: 10/15) was 6.4 months compared to 4.0 months of patients with the Gly388 genotype (events: 32/32). The HR was 0.25 (95% CI 0.12- 0.51; P = 0.0001 at Log-Rank test). No attempts were done to perform multi-variate analysis.

### Association between *FGFR4* variants, TGR, basal CEA, NLR, LDH and DC

Very few background data about genetic, clinical and/or biochemical factors predicting response to TAS-102 are available, among these, TGR, CEA, NLR and LDH at treatment start, seem the most promising. Assessments of potential factors predicting DC to TAS-102 were dichotomized: *FGFR4* p.G388R (not present vs present), TGR (low vs high), basal CEA (low vs high), NLR (low vs high), LDH (low vs high). Applied cut-offs for TGR, CEA, NLR, and LDH were 0.33%/day, 200 ng/ml, 5, and 1.2xULN U/L, respectively (Table 5). Interestingly, patients experiencing DC had more frequently an *FGFR4* p.G388R variant (*P* < 0.0001), and a low TGR (*P* = 0.0306). There were no significant associations between basal CEA, NLR, LDH values and DC to TAS-102, even if higher CEA, NLR and LDH levels were more frequent in patients whose disease was not controlled by treatment.

**Table 5:** Association between *FGFR4* p.G388R, TGRs, basal CEA, NLR and response to TAS-102

	Disease Control		
Characteristic	Yes (%)	No (%)	*P*
*FGFR4* p.G388R			
No	8 (38.1)	24 (92.3)	
Yes	13 (61.9)	2 (7.7)	< *0.0001*
TGR			
Low	14 (66.6)	9 (34.6)	
High	7 (33.3)	17 (65.4)	*0.0306*
CEA			
< 200 ng/ml	13 (61.9)	10 (38.5)	
≥ 200 ng/ml	8 (38.1)	16 (61.5)	*0.1138*
NLR			
< 5	12 (57.1)	10 (38.5)	
≥ 5	9 (42.9)	16 (61.5)	*0.2068*
LDH			
Low	12 (57.1)	8 (30.8)	
High	9 (42.9)	18 (69.2)	*0.0721*

CEA: CarcinoEmbryonic Antigen; DC: Disease Control; LDH: Lactic DeHydrogenase; NLR: Neutrophil to Lymphocyte Ratio; TGR: Tumour Growth Rate

## Discussion

In this article, we present the genetic evaluation of a cohort of clinical patients undergoing treatment with TAS-102 for extensive metastatic disease, with only 6 patients exhibiting single-organ involvement. These patients were heavily pre-treated, with 38 out of 47 having undergone 3 or 4 prior lines of therapy, and displaying poor performance status (ECOG PS 2 in 19 out of 47). Hence, TAS-102 was administered in a typical real-world clinical setting. Our findings indicate that TAS-102 is well tolerated when managed through tailored approaches, including early dose reductions, and is associated with a disease control rate of 44.7%. Given these observations, we opted to investigate any genetic characteristics that might influence response to treatment and prognosis. Indeed, gathering clinical data from routine practice offers a scientific opportunity to validate efficacy in unselected populations and to gain translational insights into specific subgroups.

Durations of responses in all patients ranged from 2 to 14 months (median: 4.5 months). Interestingly, the best outcome from TAS-102 emerged in 3 patients (NA, FA, CC) whose metastatic disease was controlled for 10, 12 and 14 months, respectively. In two of these patients, a volumetric reduction of the disease was found even if failing to meet the criteria for an objective response. Consequently, we conducted a comprehensive genetic assessment of primary tumor tissue. To the best of our knowledge, this represents the initial description of human genomic landscape characterization specifically aimed at exploring determinants of clinical benefit from TAS-102. A prevalent genetic variant, *FGFR4* p.Gly388Arg, was identified. Therefore, we focused our attention to generate hypotheses on genotype/phenotype association on *FGFR4* p.Gly388Arg.

Interestingly, it has been demonstrated that *FGFR4* p.Gly388Arg determines the exposure of a cytoplasmic signal transducer and activator of transcription 3 (STAT3) binding site. This concurs to increase the recruitment of STAT3 enhancing tyrosine phosphorylation increasing cancer progression [23]. Interestingly, *FGFR4* p.Gly388Arg was found to be correlated with worse outcomes in breast and lung cancer [24, 25], and very recently, in silico analysis in a Mexican population with colorectal cancer showed an association between rs351855 and the disease [26]. Moreover, it has been shown that trifluridine, can modulate through complex and still unknown mechanisms the phosphorylation status of ERK/AKT/STAT3 pathway [27, 28]. Taken together, our previous observations could unveil the existence of composite and complex relationships between genetic alterations in biologic pathways of FGFR4 and the effect of TAS-102. Most importantly, *FGFR4* p.G388R variant was found in patients whose disease was long-term controlled by TAS-102 and it associates with DC and prognosis. The FGFR4 pathway has been reported as a putative targetable regulator of drug resistance in CRC. In fact, silencing of FGFR4 is able to reduce CRC cell viability and it has synergistic activity with 5-fluorouracil (5-FU) and oxaliplatin chemotherapy in CRC chemotherapy-refractory cell lines by decreasing the activity of STAT3 transcription factor [29]. *FGFR4* p.G388R (rs351855) has been reported to prompt progression and affect prognosis of several cancers including CRC [30, 31]. Interestingly, this amino acid change (G > A) shows oncogenic properties by inducing tumour growth and migration of malignant cells [32]. Furthermore, this mutation in *FGFR4* has been observed alongside other gene mutations in tumor calcinosis, a rare, obscure, and debilitating disorder of phosphate metabolism characterized by the formation of hard masses in soft tissues [33]. Recently, there have been reports of a small intestine tumor and a history of surgery for lung squamous cell carcinoma. The patient subsequently developed jejunal squamous cell carcinoma, gastric adenocarcinoma, and rectal adenocarcinoma concurrently. The intestinal cancer exhibited the same rare mutations observed in the lung cancer tissue, including *FGFR4* p.G388R, suggesting a metastatic origin of the intestinal tumor from the lung [34]. The oncogenic role of the *FGFR4* p.G388R variant is further supported by a recent genetic study in a large population. Peng et al. evaluated 13,793 cancer patients and 16,179 controls and found that the G388R polymorphism is associated with increased susceptibility to cancer in the homozygous state. Stratified analysis by cancer type and ethnicity revealed similar findings for prostate cancer, breast cancer, and individuals of Asian descent [35].

In the present study, we found that *FGFR4* p.G388R is a predictive marker of response to TAS-102 in mCRC patients. This strongly suggests, in conjunction with the aforementioned previous observations, that this genetic characteristic could be a driver of tumor promotion influenced by TAS-102 administration. It is also noteworthy that *FGFR4* represents an attractive new therapeutic target in cancer treatment, and several small kinase inhibitors are in preclinical and clinical development for the treatment of various cancers, including mCRC [36]. Therefore, based on these findings, potential approaches involving the design of combinations between TAS-102 and FGFR4 inhibitors could be considered for *FGFR4*-mutated CRCs. Clinical and biochemical factors including TGR, CEA, NLR and LDH values, have been recently explored [37]. Only TGR displayed an association with TAS-102-related DC. However, although easy to assess, none of these factors can be used to precisely predict the clinical benefit from TAS-102.

Our study has some limitations. The sample size and the retrospective nature do not allow us to make definitive conclusions and to exclude any biases in patients’ selection. However, the monocentric and consecutive characteristics of the clinical cohort and the short range of patients’ accrual could limit the detrimental effects of both factors and strength its hypothesis-generating power.

## Conclusions

Our data generate the hypothesis that *FGFR4* assessment could represent a tool to rationally select patients for TAS-102 treatment and for shifting this therapy to an earlier phase of treatment. Larger studies are being planned by our group to confirm these interesting findings.

## Data Availability

The genetic data supporting the findings of this study are available upon reasonable request, which can be sent to giovanni.savarese@centroames.it. Examples of TSO500 reports are available at https://zenodo.org/records/10968790.

## Abbreviations

FDPs: Fluoropyrimidines
CRC: Colorectal cancer
TS: Thymidylate synthetase
5-FU: 5-Flurouracil (5-FU)
mCRC: Metastatic CRC
TAS-102: Trifluridine/tipiracil
ECOG PS: Eastern Cooperative Oncology Group Performance Status
UGT1: Uridine diphosphate glucuronosyl-transferase
RAS: RAt Sarcoma viral oncogene homolog
BRAF: V-raf murine sarcoma viral oncogene homolog B
EGFR: Epithelial Growth Factor Receptor
MAP: Mitogen Activated Protein
MSS: Micro-satellites status
MMR: Mismatch repair
ESMO: European Society of Medical Oncology
CT: Computerized Tomography
MRI: Magnetic Resonance Imaging
PET: Positron Emission Tomography
RX: Radiography
RECIST: Recist response evaluation criteria in solid tumors
DC: Disease control
CR: Complete response
PR: Partial response
SD: Stable disease
PD: Progressive disease
CTC: Common toxicity criteria
NGS: Next generation sequencing
FFPE: Formalin-fixed paraffin-embedded
SNVs: Small nucleotide variants
AMP: Association for Molecular Pathology
ACMG: American College of Medical Genetics
TMB: Tumor Mutational Burden
TGR: Tumour growth rate
NLR: Neutrophil-to-lymphocyte ratio
STL: Sum of target lesions
LDH: Lactic dehydrogenase
ULN: Upper limit normal
HR: Hazard ratio
CI: Confidence interval
FGFR4: Fibroblast Growth Factor Receptor 4
CEA: Carcinoembryonic antigen
Mut: Mutant
STAT 3: Signal transducer and activator of transcription 3
ERK: Extracellular signal-regulated kinase
AKT: Proetin kinase B
